# An Assessment of the Impact of Hafting on Paleoindian Point Variability

**DOI:** 10.1371/journal.pone.0036364

**Published:** 2012-05-30

**Authors:** Briggs Buchanan, Michael J. O'Brien, J. David Kilby, Bruce B. Huckell, Mark Collard

**Affiliations:** 1 Department of Archaeology and Human Evolutionary Studies Program, Simon Fraser University, Burnaby, British Columbia, Canada; 2 Department of Anthropology, University of Missouri, Columbia, Missouri, United States of America; 3 Department of Anthropology and Applied Archaeology, Eastern New Mexico University, Portales, New Mexico, United States of America; 4 Maxwell Museum of Anthropology and Department of Anthropology, University of New Mexico, Albuquerque, New Mexico, United States of America; New York State Museum, United States of America

## Abstract

It has long been argued that the form of North American Paleoindian points was affected by hafting. According to this hypothesis, hafting constrained point bases such that they are less variable than point blades. The results of several studies have been claimed to be consistent with this hypothesis. However, there are reasons to be skeptical of these results. None of the studies employed statistical tests, and all of them focused on points recovered from kill and camp sites, which makes it difficult to be certain that the differences in variability are the result of hafting rather than a consequence of resharpening. Here, we report a study in which we tested the predictions of the hafting hypothesis by statistically comparing the variability of different parts of Clovis points. We controlled for the potentially confounding effects of resharpening by analyzing largely unused points from caches as well as points from kill and camp sites. The results of our analyses were not consistent with the predictions of the hypothesis. We found that several blade characters and point thickness were no more variable than the base characters. Our results indicate that the hafting hypothesis does not hold for Clovis points and indicate that there is a need to test its applicability in relation to post-Clovis Paleoindian points.

## Introduction

Investigating the nature and causes of variation in point form is an important task for archaeologists interested in the Paleoindian period (ca. 13,600–11,450 calBP) of North America. There are two main reasons for this. One is that understanding variation in point size and shape is necessary for establishing the cultural-historical types that Paleoindian archaeologists rely on (e.g. [1]–[5]). The other is that variation in point size and shape may be informative regarding the behavior of Paleoindians, including their use of the landscape and their hunting practices (e.g. [6]–[12]).

One well-known hypothesis concerning variation in Paleoindian point form contends that it was affected by hafting. According to this hypothesis, hafting requirements constrained the size and shape of point bases but did not affect the size and shape of point blades [3], [4], [13]. An important implication of the hafting hypothesis is that the base is the most diagnostic portion of Paleoindian points [3], [4].

A key prediction of the hafting hypothesis is that base characters should be less variable than non-base characters. This prediction has been supported in several papers [3], [14]–[18], but there are reasons to be skeptical about the results of the relevant analyses. First, statistical tests were not used in the analyses, and thus it is unclear whether the differences in variability are any greater than would be expected on the basis of chance alone. Second, the analyses focused on points recovered from kill and camp sites. This is problematic because many points recovered from kill and camp sites were resharpened prior to being lost or discarded and therefore it is difficult to be sure that the differences in variability between the base and non-base portions of the points are the result of hafting constraints rather than a consequence of resharpening. Third, experimental studies using replica Clovis points suggest that both tip and base repairs would have been needed to maintain functionality [19], [20].

Given this uncertainty, we decided to re-test the hafting hypothesis. In our study, we focused on Clovis points, which are found throughout North America and are widely accepted to date to 13,600–13,000 calBP [21], [22]. We controlled for the potentially confounding effects of resharpening by analyzing points from caches as well as points from kill and camp sites. A cache is a tightly clustered deposit of artifacts that appear to have been deposited at the same time and are associated with little or no manufacturing and/or maintenance debris [23]. The majority of cached points were either not used or used only lightly before being deposited. Hence, including cached points decreases the potential for resharpening to confound tests of the hafting hypothesis. Additional differences from previous tests of the hafting hypothesis are that we used digitizing techniques to capture point form and employed a statistical test to compare the variability of the base and non-base characters.

## Materials and Methods

### 1. Sample

**Figure 1:**
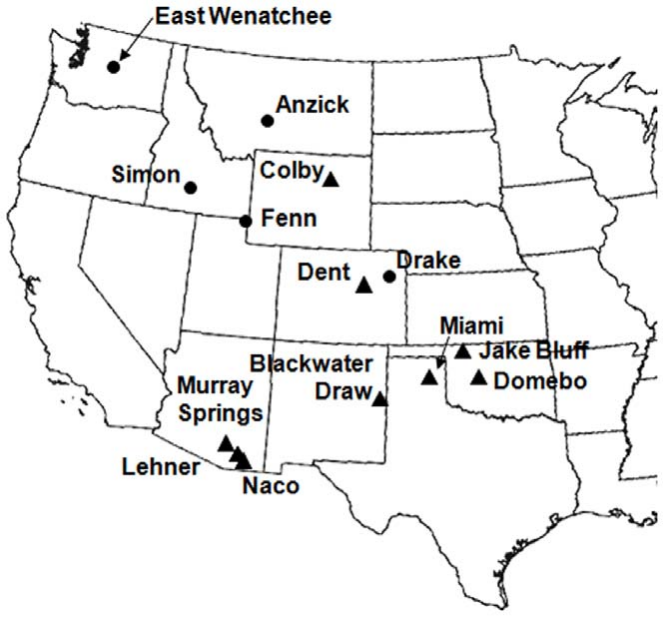
Locations of archaeological sites in the western United States from which points used in the study were recovered. Triangles = kill sites/camp sites. Circles = caches. (Figure is adapted from Buchanan et al. [71]).

Our sample comprised 122 Clovis points. We focused on complete points and specimens missing at most an ear because it is difficult to implement the data-capture methods we employed with incomplete artifacts. Sixty-eight points are from kill/camp sites and 54 are from caches. We focused on Clovis points from western North America because the distribution of Clovis caches is limited to the west. Kill/camp points come from sites located in the Southwest (Lehner, Murray Springs, and Naco), the Southern Plains (Blackwater Draw, Domebo, Jake Bluff, and Miami), and the Northern Plains (Dent and Colby). Cached points come from sites located in the Northwest (East Wenatchee, Fenn, and Simon) and the Northern Plains (Anzick and Drake). It has been suggested that the Anzick points may be burial goods rather than part of a cache, because human skeletal remains have also been recovered at the site [24]–[26]. We do not find this argument convincing for two reasons. First, the artifacts and skeleton were recovered with a front-end loader, so there is no stratigraphic evidence that they are associated [27]. Second, radiocarbon dates derived from some of the artifacts recovered at the site do not overlap with radiocarbon dates derived from some of the human bones, which suggests that they are not contemporaneous [27], [28]. Locations of the sites and the number of points per site are shown in Figure 1 and Table 1, respectively.

**Table 1:** Clovis point assemblages used in the analyses.

Site	State	Context	Number of Points*	References
Anzick	MT	Cache	6	[24]–[30]
Blackwater Draw	NM	Kill/camp	24	[31]–[36]
Colby	WY	Kill/camp	4	[37]
Dent	CO	Kill/camp	2	[38], [39]
Drake	CO	Cache	13^1^	[40]
Domebo	OK	Kill/camp	4	[41]
East Wenatchee	WA	Cache	14^2^	[42]–[45]
Fenn	UT/WY/ID^3^	Cache	16	[46], [47]
Jake Bluff	OK	Kill/camp	4	[48], [49]
Lehner	AZ	Kill/camp	10	[50]
Miami	TX	Kill/camp	3	[51], [52]
Murray Springs	AZ	Kill/camp	6	[53], [54]
Naco	AZ	Kill/camp	8	[55]
Simon	ID	Cache	5	[56]–[58]

* Number of points complete enough to be digitized.

1 Five of the points analyzed from Drake were epoxy casts.

2 We analyzed three of the points using scale drawings made by S. Moore (see [43]) and a cast of a fourth point.

3 The actual location of the Fenn cache is unknown; however, it was most likely recovered from the three-corners area where Utah, Wyoming, and Idaho meet [47].

Epoxy casts were used in lieu of some of the original points. Buchanan [59] compared casts of Clovis points from the Lehner site to the actual points and found that there was no statistical difference between the casts and the real artifacts. The paired *t*-tests he carried out gave *p* values ranging between 0.841 and 0.962. Consequently, the inclusion of epoxy casts in the sample is not expected to have affected the present study.

### 2. Data capture

**Figure 2:**
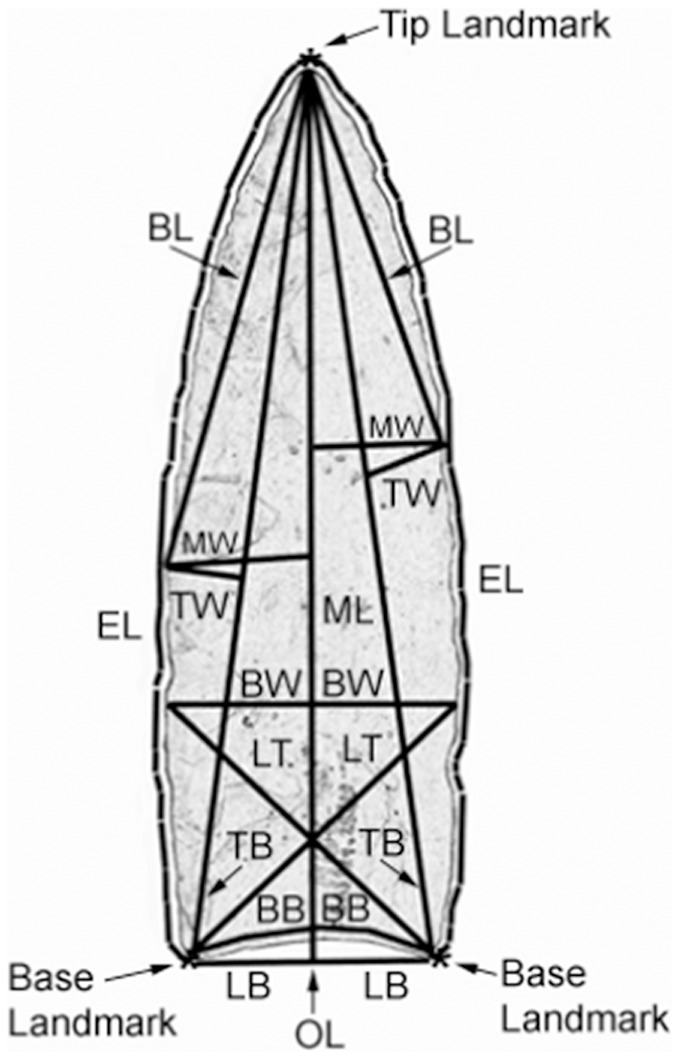
Image of a Clovis point from Blackwater Draw, NM, showing approximate location of characters. Character abbreviations follow Table 2. (Figure is adapted from Buchanan et al. [71]).

The data-capture method we used was the same as the one employed by Buchanan [59], Buchanan and Collard [6], and Buchanan and Hamilton [7]. Briefly, digital images of the points were imported into the Thin Plate Spline Digitizing Program (Version 2.02) [60]. Thirty-two landmarks were used to define the edges and base of each point, and the coordinate data were used to compute ten interlandmark distances in Matlab 6.0. The characters are listed in Table 2 and illustrated in Figure 2. In addition to the ten characters derived from digitizing the points, base thickness (BT) and maximum thickness (MT) were taken directly from points using digital calipers or were taken from published sources. Base thickness was not available for four cached points (from East Wenatchee) and seven points from kill/camp sites (four from Jake Bluff and three from Blackwater Draw). The characters were selected to capture variability in the two major parts of the points, the base and the blade, as well as variability in overall length and thickness. The characters include traditional linear measurements as well as measurements that cannot be taken accurately with calipers. Five of the characters relate to the base (BT, BB, LB, BW, and LT), three to the blade (BL, MW, and TW), and four to overall point length (ML, OL, EL, and TB). The thirteenth character, MT, is maximum thickness.

**Table 2:** Characters used in the study.

Characters	Description	Section
BB	Base boundary length. Calculated as the sum of the interlandmark distances along the nine landmarks that define the basal concavity situated between the two basal landmarks.	Base
LB	Base linear length. Calculated as the distance between the two basal landmarks.	Base
BW	Base width Width at one-third the total length above the basal landmarks.	Base
LT	Average of the right and left distances from basal landmarks to the position at one-third the total length along the opposite edge boundaries.	Base
BT	Thickness of base taken perpendicular to both basal ears.	Base
BL	Average of the right and left distances between the position of the maximum edge inflection and the tip landmark.	Blade
MW	Average of the right and left distances between the positions of the maximum edge inflections to the midline (character ML).	Blade
TW	Average of the right and left distances between the tip landmark to basal landmarks (character TB) segments to the position of the maximum edge inflection along each point edge.	Blade
ML	Midline length. Calculated as the distance from the tip landmark to the midpoint of the basal concavity (character BB).	Length
OL	Overall length. Calculated as the distance from the tip landmark to the midpoint of the segment between the basal landmarks (character LB).	Length
EL	Average of right and left edge boundary lengths. Edge boundary length is calculated as the sum of interlandmark distances along the 13 landmarks that define each edge.	Length
TB	Average of the right and left distances from the tip landmark to each of the basal landmarks.	Length
MT	Maximum thickness taken perpendicular to OL.	Thickness

The precision of the digitized characters was estimated on a sample of points from Naco and Lehner. Measurement error–the percentage of the total variance attributable to within-individual variance resulting from imprecision of measurements–was calculated for each character using Model II ANOVA [61]–[63]. Points were chosen randomly and digitized in three non-consecutive sessions, and the variance components were calculated from the resulting dataset. Measurement error associated with the characters ranges from 0.002 to 0.031 percent, which compares favorably to measurement errors reported in biological morphological studies (e.g. [61], [63]). Furthermore, there is no relationship between percent measurement error and the coefficient of variation of a character (*r* = −0.072, *p* = 0.623), which suggests measurement error does not drive variation.

We estimated missing values for nearly complete points. This was accomplished with the expectation-maximization missing-data replacement method, which uses information about covariation among variables to predict missing values [64]. A recent simulation demonstrated that this form of missing-data replacement is more precise and reliable than principal-component estimation when using a moderate number of characters (6–12) and large sample sizes [64].

### 3. Analyses

To test the prediction that base characters of Paleoindian points should be less variable than characters from other portions of points, we used the coefficient of variation (CV) and Fligner and Killeen's [65] distribution-free two-sample test (FK test). The CV, commonly used in archaeology (see refs in [66]), expresses the normalized amount of variation in a set of measurements, and is calculated by dividing the sample standard deviation by the sample mean and multiplying the quotient by 100. The FK test first ranks the CVs in the combined dataset from smallest to largest. Values that are tied are given sequential ranks. After the values are ranked, they are weighted by the sample size and then converted to the quantile of the standard normal distribution that corresponds to the weighted score. This value is then squared. Next, ties are resolved by averaging the weighted values associated with the tied values. These normalized scores are then summed to create the test statistic, *T*. Statistical significance is assessed using the large scale approximation *z*-score, which is calculated by dividing the difference between the *T* statistic and the expected *T* score by the variance. We chose the FK test to compare CVs because comparative analyses have shown that it is among the best tests for reducing type-I and type-II errors. For example, Donnelly and Kramer [67] used Monte Carlo methods and simulated data to evaluate 11 tests of relative variation measures, including a CV-based parametric bootstrap test, modifications of Levene's test, and the FK test. They found that the FK test performed best in terms of maintaining an acceptable type-I error rate when he samples were drawn from different underlying distributions, including situations where the samples had different underlying distributions. The FK test also consistently ranked as the most powerful or nearly the most powerful test in Donnelly and Kramer's [67] comparative analyses.

We carried out two analyses, one focused on kill/camp points and one on cached points. In both analyses, we used the FK test to compare the CV of each of the base characters to the CV of each of the three blade characters, the four length characters, and thickness. Because our dataset includes values for five base characters and eight non-base characters (three blade characters, four length characters, and thickness) we carried out a total of 40 FK tests in each analysis. The test prediction was that the CVs for the base characters should be significantly less than the CVs for the blade characters, the length characters, and for thickness. Both analyses were carried out in PAST version 2.00 [68]. Because we conducted multiple unplanned tests, we used Benjamini and Yekutieli's [69] method of significance-level correction. We employed this method rather than the commonly used Bonferroni correction because it has been shown to balance the reduction of type-I and type-II error rates better than Bonferroni correction [70].

## Results

The CVs for the kill/camp points are presented in Table 3. To reiterate, the hafting hypothesis predicts that the base characters should have lower CVs than the blade characters, the length characters, and maximum thickness. This is not the case. Maximum thickness is less variable than all five of the base characters; blade character MW is less variable than base characters BW, BB, LT, and BT; and blade character TW is less variable than base character BT. Thus, the qualitative comparison of the CVs for the kill/camp points does not support the hafting hypothesis.

**Table 3:** Coefficients of variation for characters of kill/camp points, ranked from smallest to largest.

Character	Section	Coefficient of Variation
MT	Thickness	21.76
LB	Base	22.08
MW	Blade	22.72
BW	Base	22.83
BB	Base	25.80
LT	Base	26.46
TW	Blade	28.96
BT^1^	Base	29.55
BL	Blade	33.04
EL	Length	35.14
TB	Length	36.19
OL	Length	36.78
ML	Length	37.41

1 Measurements of base thickness (BT) were available for only 61 of the 68 kill/camp points.

Table 4 summarizes the results of the FK tests that focused on kill/camp points. The tests indicate that the five base characters are significantly less variable than the four length characters. However, not all the base characters are less variable than the three blade characters or maximum thickness. Base characters BB and LT have CVs that are not statistically significantly different from the blade characters, and base characters LB and BT have CVs that are statistically indistinguishable from the CV for blade character MW. In addition, base character BT has a CV that is significantly greater than the CV for blade character TW, while base character BW has a CV that is not statistically different from the CVs for blade characters MW and TW. Lastly, none of the CVs for the base characters is statistically different from the CV for maximum thickness. Thus, the FK tests confirm that the kill/camp points do not support the predictions of the hafting hypothesis.

**Table 4:** Comparison of base characters (BT, BB, LB, BW, and LT) with characters describing the blade (BL, MW, and TW), different lengths (ML, OL, EL, and TB), and thickness (MT) of kill/camp points.

Section		Base	Base	Base	Base	Base
	Character	BB	LB	BW	LT	BT^1^
**Blade**	**BL**	0.0232	0.0031*	0.0039*	0.0376	0.0045*
**Blade**	**MW**	0.4840	0.3654	0.4558	0.2313	0.0904
**Blade**	**TW**	0.0359	0.0093*	0.0202	0.1044	0.0078‡
**Length**	**ML**	0.0008*	0.0001*	0.0002*	0.0016*	0.0003*
**Length**	**OL**	0.0009*	0.0001*	0.0002*	0.0018*	0.0003*
**Length**	**EL**	0.0024*	0.0003*	0.0008*	0.0045*	0.0011*
**Length**	**TB**	0.0012*	0.0001*	0.0003*	0.0028*	0.0007*
**Thickness**	**MT**	0.2411	0.3851	0.2872	0.0733	0.1233

*P*-values (one-tailed) from Fligner and Killeen's [65] distribution-free two-sample test for coefficient of variations are shown.

* Base character has CV that is significantly lesser than the non-base character using Benjamini and Yekutieli's [69] alpha correction; the critical value for 40 tests is *α* = 0.01169.

‡ Base character has CV that is significantly greater than the non-base character using Benjamini and Yekutieli's [69] alpha correction; the critical value for 40 tests is *α* = 0.01169.

1 Measurements of base thickness (BT) were available for 61 of the 68 kill/camp points.

Table 5 presents the CVs for the cached points. As before, the hafting hypothesis' prediction is that the base characters should have lower CVs than the blade characters, the length characters, and maximum thickness. The ranking of the CVs is different from the ranking yielded by the kill/camp points, but the prediction is still not supported. Base character BT is the least variable character, but maximum thickness is less variable than base characters BB, LB, and BW, and blade character MW is less variable than blade character LT. Thus, the qualitative comparison of the CVs for the cached points also does not support the hafting hypothesis.

**Table 5:** Coefficients of variation for cached points, ranked from smallest to largest.

Character	Section	Coefficient of Variation
BT^1^	Base	16.89
MT	Thickness	20.34
BB	Base	21.63
LB	Base	22.06
BW	Base	26.52
MW	Blade	28.94
LT	Base	29.01
BL	Blade	32.99
TB	Length	33.83
TW	Blade	34.01
OL	Length	34.04
ML	Length	34.11
EL	Length	34.60

1 Measurements of base thickness (BT) were available for only 50 of the 54 cached points.

Results of the cache point-focused FK tests are summarized in Table 6. As in the qualitative comparison, the results differ from the results of the equivalent analysis of kill/camp points, but the prediction is still not supported. The CVs of all the base characters are statistically indistinguishable from the CV of maximum thickness, and the CVs of base characters BB, LB, BW, and LT are statistically indistinguishable from the CVs of at least two other non-base characters. Thus, the cached points-focused FK tests confirm that the cached points also do not support the predictions of the hafting hypothesis.

**Table 6:** Comparison of base characters with characters describing the blade and point length, and thickness of cached points.

Section		Base	Base	Base	Base	Base
	Character	BB	LB	BW	LT	BT^1^
**Blade**	**BL**	0.0019*	0.0018*	0.0282	0.0932	0.0001*
**Blade**	**MW**	0.0636	0.0652	0.2100	0.3771	0.0108*
**Blade**	**TW**	0.0264	0.0293	0.0717	0.2218	0.0038*
**Length**	**ML**	0.0009*	0.0008*	0.0104*	0.0403	<0.0000*
**Length**	**OL**	0.0010*	0.0009*	0.0115*	0.0345	<0.0000*
**Length**	**EL**	0.0019*	0.0017*	0.0167	0.0435	<0.0000*
**Length**	**TB**	0.0011*	0.0013*	0.0130	0.0497	<0.0000*
**Thickness**	**MT**	0.3298	0.3240	0.1016	0.0171	0.1106

*P*-values (one-tailed) from Fligner and Killeen's [65] distribution-free two-sample test for coefficient of variations are shown.

* Base character has CV that is significantly lesser than the non-base character using Benjamini and Yekutieli's [69] alpha correction; the critical value for 40 tests is *α* = 0.01169.

1 Measurements of base thickness (BT) were available for 50 of the 54 cached points.

## Discussion

The hafting hypothesis predicts that base characters of Paleoindian points should be less variable than their non-base counterparts. The results of our analysis of Clovis points from kill/camp sites were not consistent with this prediction. While the base characters were significantly less variable than the length characters, several base characters were indistinguishable in terms of variability from the blade characters and from maximum thickness. Our analysis of cached Clovis points also did not support the prediction that base characters of Paleoindian points should be less variable than their non-base counterparts. As with the analysis of kill/camp points, the base characters were not significantly less variable than the blade characters or maximum thickness. Thus, the results of our analyses do not support the hafting hypothesis.

One issue needs to be addressed before considering the implications of our results–our choice of base characters. Two of these characters, LT and BW, might be disputed with respect to their position relative to the haft. To reiterate, character LT is the average of the right and left distances from base landmarks to the position at one-third the total length along the opposite edge boundaries, and character BW is the width at one-third the total length above the base landmarks (Figure 1). It is conceivable that the distal terminus of character LT and both termini of character BW were above the haft and thus characters LT and BW may not in fact have been constrained by the haft. We think this is unlikely. However, even if it were the case, it would not affect our findings because the other three base characters–BB, LB, and BT–undeniably relate to the part of a point that would have been hafted and are statistically indistinguishable from several non-base characters. Thus, even if characters LT and BW were rejected as base characters, our analyses would still not support the predictions of the hafting hypothesis. It appears, then, that the hafting hypothesis does not hold for Clovis points.

There are several potential reasons why the hafting hypothesis does not hold for Clovis points. One is that Clovis points were hafted in such a way that the haft did not constrain the base characters. A second possibility is that constraints were placed on the base of Clovis points, but the base was not the only portion of Clovis points that was constrained. It could be, for example, that the haft covered more of the point than imagined by proponents of the hafting hypothesis and that consequently some non-base dimensions of the point were constrained by it. Alternatively, some of the non-base dimensions may have been constrained by the demands of flight or hide-penetration, or by cultural norms. Determining which of these hypotheses is correct will require a better understanding of how large / small the dimensions of a Clovis point can be without losing functionality when different methods of hafting are used (e.g. with/without a foreshaft, with/without mastic) and when different methods of spear-delivery are employed (e.g. thrusting, unassisted throwing, atlatl-assisted throwing). One way of shedding light on this is through the replication and experimental use of spears with different combinations of Clovis points, hafts, and delivery methods (e.g. [19], [20]).

An obvious implication of our results is that it would be sensible to re-assess whether the hafting hypothesis holds for post-Clovis points. Doing so should be fairly straightforward. Earlier we pointed out that there are two potential problems with previous tests of the hafting hypothesis. One is that they did not use statistical tests. We argued that this is problematic because it means we cannot be sure the differences in variability between the base and non-base characters identified in the analyses are consequential as opposed to being simply a result of chance. The other potential problem is that the analyses focused on points recovered from kill/camp sites. We suggested this is problematic because many such points were resharpened prior to being lost or discarded, and thus it is difficult to be sure that the differences in variability are the result of hafting rather than the consequence of resharpening. Given that our analysis of kill/camp Clovis points did not support the hafting hypothesis any better than our analysis of cached Clovis points, there is reason to believe that resharpening may not in fact have undermined the results of the previous tests of the hafting hypothesis and that the real problem is the failure to use a statistical method to control for the possibility that measures of variation may differ simply by chance alone. The corollary of this is that it should be possible to revisit the previous tests of the hafting hypothesis and subject the reported measures of variation to statistical analysis. This should provide a rapid indication of whether the hafting hypothesis applies to post-Clovis points.
